# Beyond aroma: A scoping review on the impact of chronic rhinosinusitis on retronasal olfaction

**DOI:** 10.3389/falgy.2022.969368

**Published:** 2022-08-31

**Authors:** Joel James, Ilan C. Palte, Brandon J. Vilarello, Lucas G. Axiotakis, Patricia T. Jacobson, David A. Gudis, Jonathan B. Overdevest

**Affiliations:** ^1^ Department of Medical Education, CUNY School of Medicine, New York, NY, United States; ^2^ Department of Otolaryngology-Head and Neck Surgery, Weill Cornell Medicine, New York, NY, United States; ^3^ Department of Otolaryngology-Head and Neck Surgery, Columbia University Vagelos College of Physicians and Surgeons, New York, NY, United States; ^4^ Department of Otolaryngology-Head and Neck Surgery, Columbia University Vagelos College of Physicians and Surgeons, New York-Presbyterian/Columbia University Irving Medical Center, New York, NY, United States

**Keywords:** retronasal olfaction, chronic rhinosinusitis, flavor, olfactory dysfunction, olfaction, orthonasal olfaction, retronasal aroma perception

## Abstract

**Background:**

Retronasal olfaction (RNO) refers to the perception of odorants inhaled through the mouth and carried through the nasopharynx to olfactory receptors within the olfactory cleft, enabling the perception of flavor. Although orthonasal olfactory dysfunction in chronic rhinosinusitis (CRS) has been widely described, the impact of CRS on RNO is less clear. In this study, we systematically review available literature to provide an update on RNO in the setting of CRS.

**Methods:**

We systematically searched PubMed, Ovid Embase, Web of Science, and the Cochrane Library for studies examining RNO in patients with documented CRS. The primary outcome of interest was objective psychophysical measurement of olfaction, including characterization of RNO.

**Results:**

We identified 404 unique references that underwent title and abstract review by two independent reviewers, with 52 articles undergoing full-text review, where 10 relevant studies underwent data extraction. Although outcome measures varied, all included studies demonstrated diminished RNO in patients with CRS. Of six studies evaluating the relationship between retronasal and orthonasal olfactory test scores in CRS patients two out of six (33%) demonstrated a correlation between both forms of olfaction and CRS, and two out of six studies (33%) found significantly lower orthonasal olfactory test scores compared to retronasal olfactory test scores. Two of three found significant improvement in RNO with treatment of underlying CRS. Of three studies examining patient reported outcome measures (PROMs) in CRS, two found significant associations between retronasal olfactory test scores and PROMs.

**Conclusions:**

Based on the current literature, CRS patients appear to have diminished RNO, which may be associated with orthonasal olfactory dysfunction and decreased quality of life in this population. Higher level of evidence studies are required to further elucidate these relationships and the impact of medical and surgical CRS management on RNO.

## Introduction

Olfaction is a complex sensory function requiring transduction of molecular chemical odorants in the environment into neurobiological information. Olfaction can be categorized based on the path taken by odorant molecules, namely into orthonasal and retronasal olfaction. Orthonasal olfaction refers to the typical sensation of “smell,” whereby odorants pass through the nares during inhalation to reach receptors in the olfactory cleft (OC). These receptors interpret the presence of these molecules into a perceived odor. In contrast, retronasal olfaction (RNO) refers to the passage of odorant molecules within the mouth that travel through the posterior nasopharynx en route to olfactory receptors (1). Information transduced by retronasal function contributes to the perception of flavor (1). Retronasal and orthonasal function are distinct physiologic processes, with evidence pointing to divergent mechanisms of neurobiological processing (2). Both retronasal and orthonasal olfactory dysfunction (OD) have been shown to diminish quality of life, especially when identification of food is adversely impacted (3).

Olfactory assessment may be performed using subjective self-report methodology or semi-objective psychophysical assessment. Psychophysical testing is most commonly performed for orthonasal olfaction, where frequently used testing options include a microencapsulated odorant format in the 40-item Smell Identification Test (SIT) or 12-item Brief Smell Identification Test (BSIT) (4) or a marker based platform using “Sniffin” Sticks” or “Snap and Sniff” odorant markers that assess participant olfaction in the domains of threshold, discrimination, and identification of odorants (5, 6). Retronasal olfactory testing is less frequently evaluated, however, commonly used methods include the candy smell test, and “tasteless” powders (7). These assessments use flavored candies (8), taste stimulants, such as spices and instant drinks in powder form (9) or “tasteless” powders (10) when asking participants to identify an associated odor. Use of these testing methods has been important in comprehensively determining the extent of olfactory dysfunction in patients.

Chronic rhinosinusitis (CRS), a condition of persistent sinonasal inflammation, is known to cause OD. The relationship between chronic rhinosinusitis and orthonasal OD is well documented in the literature, where studies suggest decreased olfaction-related quality of life in CRS patients, with positive correlation between olfactory improvement and resolution of CRS symptoms (11). Evidence of the impact of CRS on retronasal OD, remains less clear. The studies identified in the literature primarily use a combination of psychophysical tests to evaluate retronasal and orthonasal OD, surveys of patient quality of life, and imaging of the olfactory cleft; however, variation in methodology impairs comparative assessment.

In this study we aim to systematically survey the literature to clarify the relationship between RNO and CRS, including the differential impact on retronasal compared to orthonasal olfaction, radiologic evidence for retronasal olfactory dysfunction, and improvement of RNO with currently available CRS treatment modalities. Specifically, in a population of adults with CRS, we aim to explore RNO psychophysical testing scores, quality of life scores, and their comparison to orthonasal olfaction.

## Methods

In accordance with the Preferred Reporting Items for Systematic Reviews and Meta-Analyses extension for Scoping Review guidelines (PRISMA-ScR) (12), we performed systematic search queries in PubMed, Ovid Embase, Web of Science, and the Cochrane Library along with bibliographic review in consultation with a research librarian at Weill Cornell Medicine to identify retronasal olfactory function studies from inception until February 2022.

The search queries consisted of a combination of subject headings and keywords grouped by the following concepts: retronasal olfaction and chronic rhinosinusitis. Subject headings and keywords for each concept were combined using Boolean operators. Pubmed was searched using the following search string: (retronasal OR gustatory OR “olfactory flavor” OR “olfactory dysfunction”) AND (sinusitis[MeSH] OR “Chronic Rhinosinusitis” OR “Nasal Polyps"[MeSH] OR “nasal polyp” OR “nasal polyps” OR “Chronic sinusitis”). The full list of search queries is contained in Supplementary Appendix A.

Inclusion criteria included peer-reviewed studies of any design primarily investigating RNO in an adult population with chronic rhinosinusitis using objective psychophysical assessments. Articles not published in English, lacking objective RNO measurement, or lacking a patient population with chronic rhinosinusitis, and conference abstracts were excluded. Two reviewers (JJ and IP) independently conducted the initial title and abstract review. The full texts of all records passing the initial screening were retrieved to confirm final eligibility by two independent reviewers (JJ and IP). Bibliographies of included studies and systematic reviews were reviewed to confirm comprehensiveness of article inclusion. A third author (BV) independently reviewed any discrepancies.

Articles were classified based on themes that emerged during full-text review of the articles. The primary outcome extracted was objective psychophysical measurement of RNO. Other extracted data included author, year, study design, primary and secondary outcomes of the included studies, and a summary of the findings.

## Results

**Figure 1:**
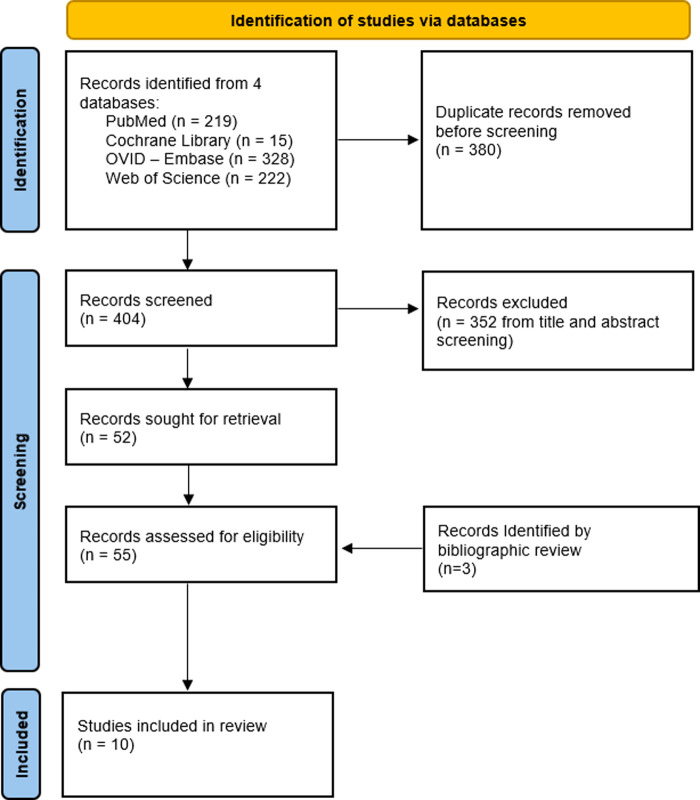
PRISMA-Style flow diagram of study retrieval and selection.

Figure 1 provides a PRISMA-style flow diagram of study retrieval and selection. Unique references identified by the search criteria included 404 entries that underwent title and abstract review, which resulted in 52 articles retained for full-text review. Of these 52 articles, 45 studies were subsequently excluded. Three additional studies were identified through bibliographic review, resulting in a total of 10 studies ultimately undergoing data extraction.

Of 10 included studies, eight were cross-sectional in design, one was a randomized control-trial, and one study was a descriptive case report. Two studies used the Candy Smell Test (CST) (8), a validated tool for measure of RNO and eight studies obtained RNO data through variations of a test involving the identification of a predetermined set of odorants placed in the oral cavity, as previously described in the literature (9). Characteristics of included studies are demonstrated in Table 1.

**Table 1:** Characteristics of included studies.

Author	Year	Study Design	Sample	Primary Outcome	Secondary Outcome (s)	Method of RNO Measurement	Notable Findings
Zhao et al.	2006	Case Report	49yo F w/ CRSwNP	Orthonasal and retronasal odor threshold and identification at different stages in CRS treatment	Change in nasal airflow dynamics before and after surgical treatment	Retronasal odor identification of odorant presented in a cap placed in oral cavity	Improved retronasal olfaction post-surgery *via* improvement of mechanical obstruction.
Rombeaux et al.	2009	Observational case series	Patients with olfactory disorders at outpatient clinics	Sniffin’ Sticks TDI score and retronasal odor identification	Olfactory-related event potentials and trigeminal event-related potentials to etiology of OD	Odorized powder presented to oral cavity	No differences in orthonasal and retronasal in CRS. CRS patients had more retronasal OD than healthy, but less retronasal OD than patients with other olfactory disorder etiologies.
Othieno et al.	2018	Cross-sectional	Patients with CRS who had not undergone surgery	Sniffin’ Sticks TDI score, retronasal odor identification, and Olfactory Cleft Endoscopy Score (OCES)	Patient reported outcome measures: Sino-Nasal Outcome Test (SNOT-22), Questionnaire of Olfactory Disorders (QOD-NS), Chemosensory Complaint Score	Odorized powder presented to oral cavity	CRS patients demonstrated deficits in RNO, with worse scores in patients with nasal polyposis, asthma, and AERD. RNO scores correlate with OCES, QOD-NS, and CCS scores and smell/taste questions of SNOT-22.
Liu et al.	2020	Cross-sectional	Patients with CRS undergoing surgery who underwent paranasal CT for surgical planning	Radiologic opacification of olfactory cleft in 2D and 3D reconstruction	Orthonasal odor TDI score and retronasal odor identification	Candy Smell Test	Increased OC opacification is associated with worse RNO scores.
Landis et al.	2003	Cross-sectional	Patients with CRS with newly discovered NP who had not received treatment	Orthonasal odor identification	Retronasal odor identification	Odorized powder presented to oral cavity	Orthonasal OD was more pronounced than retronasal OD in patients with CRSwNP.
Ganjaei et al.	2018	Cross-sectional	Patients from tertiary rhinology clinic with CRSwNP or CRSsNP	Sniffin’ Sticks TDI and retronasally presented odorants		Odorized powder presented to oral cavity	Retronasal and orthonasal identification of most odors correlate in CRS patients; however, patients with anosmia can still identify certain retronasal odors more often than expected.
Besser et al.	2021	Cross-sectional studies	Patients with CRS (with and without nasal polyps) who underwent surgical treatment	Sniffin’ Sticks TDI score and retronasal odor identification	Patient-reported outcome measure surveys (SNOT-20 and ETDQ-9)	Candy Smell Test	Those with CRSwNP had lower CST scores than those with CRSsNP, no significant improvement post-operatively.
Rowan et al.	2018	Cross-sectional studies	Adult patients with CRS at tertiary rhinology practice	Patient-reported eating-related quality of life survey (QOD-NS)	Orthonasal odor TDI score, retronasal odor identification, and taste identification	Odorized powder presented into oral cavity	Patients with AERD and those with self-reported impaired eating QoL have worse RNO.
Reychler et al.	2014	Randomized control trial	Patients with CRS with or without NP who had not undergone surgery	Sniffin’ Sticks TDI score	Retronasal odor identification	Odorized powder presented into oral cavity	Treatment of CRS with oral spray, nebulized corticosteroids, or nasal spray improved retronasal olfaction, although one method was not superior to the other. Orthonasal and retronasal olfactory scores were not correlated.
Rombeaux et al.	2006	Cross-sectional studies	Patients with normal olfaction, nasal polyposis, posttraumatic olfactory loss, and postinfectious olfactory loss	Sniffin’ Sticks TDI score, retronasal odor identification	Magnetic Resonance Imaging (MRI) of olfactory bulb, chemosensory electrophysiology recordings	Odorized powder presented into oral cavity	In nasal polyposis, orthonasal olfactory function is significantly more impaired than RNO function.

### Retronasal olfaction in CRS

Across all 10 studies (100%), patients with CRS were found to have diminished objectively measured retronasal olfactory test scores. Notably, two studies included healthy controls in their study design, where the control patients demonstrated higher RNO scores compared to patients with documented CRS with nasal polyposis (CRSwNP) (13, 14). Six studies (60%), found diminished RNO in CRS cohorts without direct comparison to healthy controls (15–20). One study found that patients with CRSwNP, CRS without nasal polyposis (CRSsNP), and non-eosinophilic CRS all had CST scores indicative of retronasal OD (21). Besser et al. found CST scores to be indicative of retronasal OD across all included CRS patients (22).

### Retronasal olfaction in CRS subtypes

Four studies were identified that compared differences in RNO among CRS subtypes (Table 2) (16, 18, 21, 22). In three studies, those with CRSwNP had significantly lower RNO scores than those with CRSsNP (16, 18, 22). Notably, in one study, the degree of RNO impairment was more significant in those with aspirin-exacerbated respiratory disease (AERD); however, the significance of this association was lost in multivariable regression analyses when accounting for the degree of endoscopic OC inflammation (18). In one study, CST scores were not significantly different among patients with CRSwNP, CRSsNP, and non-eosinophilic CRS (21).

**Table 2:** Retronasal olfactory test scores by CRS subtypes.

Author	Year	Retronasal Olfaction Measure	CRSsNP	CRSwNP
Liu et al.	2020	Candy Smell Test	17.9 ± 3.2	14.1 ± 6.4
Besser et al.	2021	Candy Smell Test	18.0 ± 3.4	13.4 ± 6.4*
Ganjaei et al.	2018	Odorized powder presented to oral cavity	10.28 ± 4.08	13.31 ± 3.50*
Othieno	2018	Odorized powder presented to oral cavity	13.2 ± 3.5	10.2 ± 4.1**

* *p* < 0.05.

** *p* < 0.01.

### Retronasal vs. orthonasal olfactory function in CRS

Six studies explored the relationship between retronasal and orthonasal olfactory function in CRS (Table 3) (13, 14, 16, 18–20). Two studies found retronasal and orthonasal olfactory function scores to be correlated in CRS patients (16, 18). In one study, retronasal olfaction scores were significantly correlated with both total Sniffin' Sticks Threshold, Discrimination, and Identification (TDI) scores and individual TDI component subscores in patients with CRS. When using TDI cutoffs for anosmia, hyposmia, and normosmia, statistical differences in olfactory perception were shared across orthonasal and retronasal olfaction assessment scores (18). In another study, RNO scores were associated with orthonasal Sniffin' Sticks scores for discrimination and identification, but not threshold. In this study, certain odorants common to both tests were identified more often through the retronasal than the orthonasal route (16). Although both types of olfactory function were diminished compared to healthy controls, two studies found patients with nasal polyposis had significantly higher RNO scores than orthonasal TDI scores, indicative of relative RNO preservation and more severe orthonasal OD (13, 14). One study found no association between RNO testing and orthonasal Sniffin' Sticks TDI scores in a patient cohort with CRS-related OD (20). In one randomized-controlled trial that measured the impact of corticosteroid administration route (oral, nasal spray, and sonically nasally nebulized) on olfaction in patients with CRS, the retronasal olfactory test and orthonasal Sniffin' Sticks TDI scores were not related, neither before, nor after treatment (19).

**Table 3:** Comparison between orthonasal and retronasal olfactory test scores in patients with CRS.

Author	Year	Orthonasal Sniffin’ Sticks TDI	Retronasal Olfaction Assessment*
Rombaux et al.	2006	Normal olfactory function: 33.9 Nasal polyposis: 12.3	Normal olfactory function: 18.5 Nasal polyposis: 14.9
Rombaux et al.	2009	CRS: 18.5	CRS: 13.1
Rowan et al.	2018	Normal eating QOL: 25.4 ± 8.5 Impaired eating QOL: 15.1 ± 6.4	Normal eating QOL: 12 ± 4.3 Impaired eating QOL: 9.6 ± 3
Recyhler et al.	2014	Pre-treatment oral systemic: 22.2 ± 3.7 Pre-treatment nasal spray: 24.4 ± 4.2 Pre-treatment sonic: 20.2 ± 9.6 Post-treatment oral systemic improvement: 5.8 ± 4.1 Post-treatment nasal spray improvement: −1.1 ± 3.3 Post-treatment sonic improvement: 5.5 ± 7.5	Pre-treatment oral systemic: 13.7 ± 3.9 Pre-treatment nasal spray: 15.3 ± 3.6 Pre-treatment sonic: 11.9 ± 3.4 Post-treatment oral systemic improvement: 4.2 ± 4.7 Post-treatment nasal spray improvement: 0.7 ± 2.4 Post-treatment sonic improvement: 1.1 ± 6.6
Othieno et al.	2018	N/A	CRSwNP: 10.2 ± 4.1 CRSsNP: 13.2 ± 3.5
Ganjaei et al.	2018	CRSsNP: 20.76 ± 9.35 CRSwNP: 26.3 ± 18.11	CRSsNP: 10.28 ± 4.08 CRSwNP: 13.31 ± 3.50
Landis et al.**	2003	CRSwNP: 5.1/10 Healthy Controls: 8.4/10	CRSwNP: 6.4/10 Healthy Controls: 8.5/10

* Clinical retronasal olfactory function assessment originally described in Heilmann et al. 2002 (7), exact methods varied slightly among each study.

** This clinical test used only 10/16 odorized Sniffin’ Sticks in the identification test only (no discrimination or threshold data included).

### Radiologic/endoscopic findings and RNO in CRS

One study utilized pre-treatment paranasal computed tomography of patients with CRS to evaluate the role of OC opacification with RNO. In this study, complete OC opacification was associated with lower CST RNO scores when compared to partial OC opacification (21). One study found a significant negative correlation between RNO scores and Olfactory Cleft Endoscopic Scale (OCES) score, a validated endoscopic grading system (18, 23).

### Response of RNO to treatment of CRS

Two studies detailed the response of RNO to surgical intervention for CRS (17, 22). In one descriptive case report, a patient with CRSwNP was found to have improved retronasal odor identification following endoscopic sinus surgery (ESS) with removal of incident polyps (17). Another study found no statistically significant change in CST scores among patients with CRS tested pre- and post-ESS (22).

In a randomized controlled trial analyzing the effect of the route of corticosteroid administration on RNO in patients with CRS, oral administration and sonically nebulized administration of corticosteroids showed statistically significant improvement in orthonasal scores compared to nasal administration, but all three delivery modalities had similar improvements in RNO (19).

### RNO and patient reported outcome measures (PROMs) in CRS

We identified three studies that explored the relationship between RNO and PROMs in CRS (15, 18, 22). In one study, RNO identification scores correlated with olfactory-related quality of life as measured by the Questionnaire of Olfactory Disorders (QOD-NS), including correlations between all subdomains of the QOD-NS. RNO scores also correlated with the smell/taste question of the 22-item Sinonasal Outcome Test (SNOT-22) and the Chemosensory Complaint Score (CCS) smell subdomain. However, orthonasal scores were more strongly correlated with PROMs than retronasal scores. RNO scores were correlated with neither total SNOT-22 nor CCS taste scores in this study (18). Another study found no association between CST scores and both the subjective assessment of flavor and SNOT-22 scores (22). In a study examining the association between impaired eating-related quality of life, as measured by QOD-NS and CCS taste scores and olfactory and gustatory dysfunction, patients with impaired eating-related quality of life were found to have significantly worse retronasal identification scores than CRS patients with a normal eating-related quality of life; however, these findings were not significant in multivariable analyses (15).

## Discussion

### Summary of findings

In this scoping review, we survey the available literature examining the impact of CRS on RNO. Included studies demonstrated impaired RNO in patients with CRS, particularly in those with nasal polyposis and OC opacification. Additionally, quality of life appears to be diminished in those with CRS and OD. Despite these findings, there is a compelling need to better understand the relationship between CRS, the treatment of associated symptoms, and patient-reported outcome measures and objective measurements of RNO.

### Drivers of retronasal OD in CRS

Retronasal olfaction is dependent on the movement of air from the mouth to the olfactory receptors in the OC *via* the posterior nasopharynx (1). Retronasal OD has been postulated to be related to the impairment of airflow in the setting of obstruction, inflammatory pathologies, or direct OC neuroepithelium inflammation (24). In CRS, physical barriers caused by anterior obstruction of the nose and opacification of the OC are likely due to the edema, crusting, and inflammation of the nasal mucosa; all factors contributing to orthonasal OD in CRS (25, 26). On review of the literature, it appears that OCES scores indicative of worsening OC inflammation and OC opacification on imaging are associated with both retronasal and orthonasal OD, while anterior obstruction of the nose, such as the presence of nasal polyposis may contribute more substantially to orthonasal OD in the setting of sustained RNO (13, 14, 18, 21). One study evaluating olfaction with artificial obstruction of the anterior nose illustrated impaired orthonasal olfaction with relatively preserved RNO in healthy patients (27). A subsequent study modeling anterior and posterior nasal cavity obstruction in healthy patients reported impairment in both orthonasal and retronasal olfaction, demonstrating the importance of choanal patency in RNO (28), where the location of nasal polyposis also has a differential impact on the degree of OD (29). These findings may contribute to the variability when comparing orthonasal and retronasal OD outcomes in CRS. Thus, while OC inflammation drives both retronasal and orthonasal OD due to its independence from airflow directionality, anterior polypoid obstruction may play a more important role in orthonasal OD than retronasal OD due to preservation of retronasal air flow among those with CRSwNP. Routine assessment of OC and location of polyposis on imaging may provide valuable insight into the presence OD in CRS patients and augment psychophysical testing of olfactory function.

### Response of retronasal OD to CRS treatment

The studies evaluated in this review also provide insight into how various CRS treatment modalities impact retronasal OD. Notably, nebulized and orally administered corticosteroids improved orthonasal olfaction more than nasally administered corticosteroids but all three modalities similarly improved RNO (19). Previous studies have shown standard pressurized intranasal corticosteroids have decreased distribution to the superior turbinate, OC, and sphenoethmoidal recess with a high proportion of delivery occurring in the anterior third of the nose (30–32). Conversely, nebulized corticosteroids saturate the air that is inhaled, leading the corticosteroid to be delivered to any interface that is usually encountered by inhaled air, including the OC. Thus, the more effective delivery of anti-inflammatory corticosteroids to the OC may differentially improve OD compared to administration of corticosteroids that fails to reach the OC. Orally administered corticosteroids may effect a similar change in RNO due to the systemic delivery of the medication, including delivery to the OC. Further research investigating retronasal OD response to other CRS treatments, such as biologics, sinus surgery, and antibiotics, could contribute meaningfully to the understanding of CRS therapy and pathology of retronasal OD in CRS.

### PROMs and RNO scores

Previous work has suggested that those with retronasal OD may be less aware of their smell loss compared to those with orthonasal OD and may not necessarily endorse decreased flavor perception and quality of life (33). Interestingly, the studies included in this review found varied relationships of RNO to PROMs in CRS; some studies found an association with PROMs and QoL, although a greater association was found between orthonasal OD and CRS PROMs. One study postulated that orthonasal OD may have a greater contribution to perception of flavor than previously understood, particularly through flavor anticipation (13). Others have proposed that flavor perception is mediated by unconscious memory recall from previously experienced cross-modal sensory interactions making retronasal OD less noticeable (18, 33). Other theories include increased compensation by other components of flavor such as texture and gustatory function, which in turn allow for retrieval of memories associated with food or drink (34, 35). Interestingly, one study found that QoL in patients with sinonasal complaints is more dependent on orthonasal than retronasal olfaction; RNO was found to play a larger role in QoL reduction for other causes of anosmia (3). This may be due to the differences in etiology of OD in these patients, in line with the finding in our review that orthonasal OD may cause more significant distress in CRS. Further research is required to explore the relationship between the differential impact of orthonasal and retronasal OD on olfaction-related PROMs in CRS, particularly in the experience of food and perception of flavor.

This study is not without limitations. Most included studies in our review were cross sectional in design, yielding lower level of evidence. Additionally, there is not a high volume of studies investigating RNO in CRS in the literature. An increased volume of higher level-of-evidence studies will meet the need demonstrated in this review. More ubiquitous RNO psychophysical testing, measurement of PROMs, and correlation with endoscopic and radiologic findings will pave the way for a clearer, more complete understanding of RNO in CRS.

## Conclusion

Current literature suggests that CRS is associated with retronasal OD, particularly in those with opacification and inflammation of the OC. However, the association between orthonasal and retronasal OD as well as how each may be impacted by duration of CRS symptoms and presence of nasal polyps remains vague. Higher level of evidence studies are needed to further characterize RNO in CRS phenotypes and endotypes, the relationship between retronasal and orthonasal OD in CRS, quality of life in those with CRS and retronasal OD, and response to currently-available CRS treatment.

## Data Availability

The original contributions presented in the study are included in the article/Supplementary Material, further inquiries can be directed to the corresponding author/s.
