# Supplementary material for: Beyond aroma: A scoping review on the impact of chronic rhinosinusitis on retronasal olfaction
Source: Front Allergy. 2022 Aug 31;3:969368. doi: 10.3389/falgy.2022.969368 (PMC9470759; doi:10.3389/falgy.2022.969368)
Supplement: Supplementary file 1 [file Table_1_v2.docx]

**Search Terms for Scoping Review on Retronasal Olfaction in Chronic Rhinosinusitis**

| **Database** | Search | **Hits** |
| --- | --- | --- |
| **Pubmed** | (retronasal OR gustatory OR "olfactory flavor" OR "olfactory dysfunction") AND (sinusitis[MeSH] OR "Chronic Rhinosinusitis" OR "Nasal Polyps"[MeSH] OR "nasal polyp" OR "nasal polyps" OR "Chronic sinusitis") | **219** |
| **Web of Science** | 1 TS=(retronasal OR gustatory OR "olfactory flavor" OR "olfactory dysfunction")  2 TS=("Chronic Rhinosinusitis" OR "nasal polyp" OR "nasal polyps" OR "Chronic sinusitis")  3 #1 AND #2 | **222** |
| **Ovid Embase** | 2: Retronasal.mp.  3: gustatory.mp  4: olfactory flavor.mp  5: olfactory dysfunction.mp  6: 2 or 3  7: 4 or 5  8: 6 or 7  9: sinusitis.mp  10: rhinosinusitis.mp  11: nasal polyps.mp  12: nasal polyp.mp  13: chronic sinusitis.mp  14: 9 or 10  15: 11 or 12  16: 14 or 13  17: 15 or 16  18: 8 and 17 | **328** |
| **Cochrane** | ((retronasal OR gustatory OR "olfactory flavor" OR "olfactory dysfunction") AND ("Chronic Rhinosinusitis" OR "nasal polyp" OR "nasal polyps" OR "Chronic sinusitis")):ti,ab | **15** |
